# The pathogenic mechanism of oral bacteria and treatment with inhibitors

**DOI:** 10.1002/cre2.499

**Published:** 2021-10-09

**Authors:** Thuraya Elgreu, Sean Lee, Sabrina Wen, Radwa Elghadafi, Thanarut Tangkham, Yun Ma, Bing Liu, Serge Dibart, Xiaoren Tang

**Affiliations:** ^1^ Henry M. Goldman School of Dental Medicine, Department of Periodontology Boston University Boston Massachusetts USA; ^2^ Department of Corporate Finance and Accounting Bentley University Waltham Massachusetts USA; ^3^ Henry M. Goldman School of Dental Medicine, Department of General Dentistry Boston University Boston Massachusetts USA

**Keywords:** cytokine(s), cell signaling, inflammation, periodontal medicine

## Abstract

**Objectives:**

The objective of this study was to introduce the evidence obtained through extensive research that periodontitis increases risk of many systemic diseases.

**Method:**

Analysis of some oral bacteria (P. gingivalis, T. denticola, T. forsythia, A. actinomycetemcomitans, and F. nucleatum) and its related treatments and mediators by the specific methods (western blot, ELISA, etc).

**Results:**

This article reviews in detail the evidence obtained through extensive research that periodontitis increases risk of many systemic diseases, including cardiovascular disease, rheumatoid arthritis, and Alzheimer's disease. These diseases are known to be associated with some certain specific gram‐negative bacteria as periodontal pathogens, which induce inflammation and related diseases through TLR receptors, kinases, transcriptional factors and other cytokines. We also reviewed the latest research for inhibitors against inflammation and related diseases that have potential to be further applied clinically. In addition, based on a large amount of research evidence, we draw two tables about the mechanism of disease caused by periodontal bacteria, so that readers can easily search and analyze these research results.

**Discussion:**

This review details how the periodontal bacteria and their virulence factors can trigger host immune defense and induce many systemic diseases via inflammation and invasion. This Review also addressed the latest research around inhibitors against inflammation.

## 1 INTRODUCTION

The link between dental diseases and systemic diseases has long been a controversial subject, which can be traced back to ancient Greece when Hippocrates reported a cure of arthritis after teeth extraction. During the early to mid‐20th century, the “focal infection theory” was widely accepted, which claimed that localized infections cause diseases elsewhere in the body, with oral infection being the prime focus. Many therapeutic edentulations were performed during this period even though that there was no convincing evidence pinpointing teeth to be the “foci” of infection. The focal infection theory era ended around 1950 after researches showed that teeth extraction did not consistently cure diseases, such as rheumatoid arthritis. However, the revival of the focal infection theory started to gain momentum in the 1990s after researches linked periodontal disease to atherosclerotic vascular disease. Since then, emerging evidence has shown that periodontitis potentially increases the risk of many systemic diseases, including cardiovascular disease, rheumatoid arthritis, and Alzheimer's disease (Holmer et al., 2018).

Periodontitis is an inflammatory disease caused by a host reaction to a mixed bacterial infection. Out of 700 bacterial species, some specific bacteria, such as *Porphyromonas gingivalis*, *Treponema denticola*, *Tannerella forsythia*, *Aggregatibacter actinomycetemcomitans*, and *Fusobacterium nucleatum* are regarded as the principal periodontal pathogens. They play an important role in the occurrence and development of periodontal diseases and other diseases (Kim & Amar, 2006).

The causes behind periodontal pathogenic inflammation and other related systemic diseases have become increasingly intriguing and complicated. A large number of studies have found that periodontal pathogens transmit their signals into animal cells or tissues through cell receptors, including Toll‐Like receptors 2/4 (TLR2/4), induce phosphorylation of related kinases, express transcriptional factors, regulate cytokines (Jia et al., 2019), and finally induce various diseases (Kim & Amar, 2006). However, a few questions remain: How do these major periodontal pathogens transmit signals through cell receptors such as TLR2/4? Why do the signals of these pathogens induce different factors, cytokines, and diseases, even though they go through the same receptors? To answer these questions, it is necessary to understand this through the following review on how periodontal bacteria induce diseases.

With a deeper understanding of the mechanisms of how pathogens infect animals and cause inflammation and disease (Hasturk & Kantarci, 2015), there will be more opportunities for us to develop effective inhibitors or drugs. So far, a large number of inhibitors or drugs specific to suppression of periodontal pathogens have been developed (Maksylewicz et al., 2019). Since the information about these inhibitors has been in disarray, we provide the following review on inhibitors used for periodontal bacteria‐induced Inflammation.

## 2 CELLS AND METHODS

The oral bacteria, mammalian cells, and the experimental methods cited below should be approved by the related school's Institutional Animal Care and Use Committee if they are involved in human subjects or vertebrate animals (reference below for examples of the different cells and methods that have been individually used). Examples of oral bacteria and mammalian cells include: *P*. *gingivalis*, *T*. *denticola*, *T*. *forsythia*, *T*. *denticola*, *A*. *actinomycetemcomitans* cells, neutrophils, macrophages, primary bone marrow macrophage, endothelial cells, brain cells, or tumor cells. Examples of methods include: A treatment of cells with lipopolysaccharides (LPS), protein array, enzyme‐linked immunosorbent assay, and western blot analysis, and so forth.

## 3 MAJOR PERIODONTAL BACTERIA

In 1998, Socransky et al. 1998 classified microbials in subgingival plaque into multiple complexes based on cluster and ordination analysis. The red complex consisted of *P*. *gingivalis*, *T*. *denticola*, and *T*. *forsythia* are gram‐negative late colonizers that relate closely to clinical measurements of periodontal disease. The name, *P*. *gingivalis*, originates from the Greek word porphyromonas (which means purple) due to its hemin forming property when growing on blood agar plates. Their fimbriae promote aggregation and induces pro‐inflammatory cytokine expression. The outer membrane sheds evaginations (outer membrane vesicles) into the environment. These vesicles contain some of the most important virulence factors of *P*. *gingivalis*: Gingipains, and LPS. Gingipains are cysteine proteases that cleave many host proteins to allow *P*. *gingivalis* to escape the immune system and weaken the inflammatory response. In addition to directly degrading cytokines such as tumor necrosis factor‐alpha (TNF‐α) and interleukin‐1 beta (IL‐1β), some of its other well‐known functions are to inhibit the phosphoinositide 3‐kinases (PI3K)/protein kinase B (AKT) pathway, reduce cluster of differentiation 14 (CD14) expression, and cleave complement component 5 (C5) into C5a and C5b, which further leads to a complement‐TLR crosstalk that suppresses the immune response. On the other hand, LPS elicits the immune response through binding with TLR‐2 and TLR‐4. Moreover, there have been reports of crosstalk between TLRs‐2, 4, and 9. TLRs have a TIR domain, which is responsible for signal transduction. Myeloid differentiation primary response 88 (MyD88), TIR‐domain‐containing adapter‐inducing interferon‐β (TRIF), and TIRAP are toll/interleukin‐1 receptor (TIR) domain‐containing adaptor proteins downstream of TLRs, which further activates signaling pathways such as nuclear factor kappa B (NF‐κB), Activator protein 1 (AP‐1), mitogen‐activated protein kinase (MAPK), c‐Jun N‐terminal kinases (JNK), PI3K, or extracellular signal‐regulated kinases (ERK) (Jia et al., 2019). *P*. *gingivalis* also produces peptidylarginine deiminase (PADs) and heat‐shock proteins (GroEL), which have both been linked to non‐oral diseases (Lin et al., 2015).

*T*. *denticola* was originally described as “spirochaete denticola” and later proposed as a distinct species “*T. denticola*” (Chan et al., 1993) after three strains were isolated from deep periodontal pockets. Studies have indicated that *T*. *denticola* triggers the immune response through TLR2 and its periplasmic flagella is crucial for this stimulation (Ruby et al., 2018). The outer membrane, or more commonly known as the outer sheath, hosts major sheath proteins (MSP), and lipooligosaccharide (LOS). They both exhibit the ability to induce pro‐inflammatory cytokines. A trademark virulence factor of *T*. *denticola* is its prolyl phenylalanine‐specific peptidase (dentilisin), which can degrade host cell proteins and activate polymorphonuclear neutrophils (PMNs) via the complement C3 pathway (Yamazaki et al., 2006).

*T*. *forsythia* was originally referred to as *Bacteroides forsythus* and later renamed due to it being discovered by Dr. Anne Tanner of the Forsyth Institute. A unique surface layer formed of glycosylated proteins covers the outer membrane of *T*. *forsythia* and is responsible for adhesion to and invasion of cells and coaggregation with other species. It also attenuates pro‐inflammatory cytokine expression by evading immune recognition. BspA, a cell surface‐associated and secreted protein can induce chemokine expression through binding with TLR2. Its LPS has also been associated with inflammation. Another significant periodontal pathogen is *A. actinomycetecomitans*, which is considered to be an early colonizer and has been found to be highly associated with localized aggressive periodontitis, as well as chronic periodontitis. Its virulence factors LPS, leukotoxins, and cytolethal distending toxins (Cdts) can all induce inflammation (Belibasakis et al., 2019). Leukotoxins plays a crucial role in the development of aggressive periodontitis through its specificity to macrophages and polymorphonuclear cells (PMNs) via binding with lymphocyte function‐associated antigen 1 (LFA‐1) CD18 subunit, leading to suppressed immune response. Furthermore, leukotoxins can hyperactivate hosts (Gómez‐Bañuelos et al., 2019).

*F*. *Nucleatum* is also an important periodontal pathogen due to its ability to coaggregate with other species, therefore, playing a key role in the formation of dental plaque. It has received attention from the scientific community due to its association with multiple diseases such as adverse pregnancy outcomes and gastrointestinal disorders. It produces virulence factor FadA, which is considered an adhesin/invasin has been hypothesized to be responsible for both a direct invasion of host cells and increased endothelial permeability for other bacteria invasion (Fardini et al., 2011).

## 4 MECHANISM OF MAJOR PERIODONTAL BACTERIA INDUCED‐INFLAMMATION AND ASSOCIATED DISEASE

Ever since Beck et al. published an article linking periodontal and cardiovascular disease in 1996, there has been increasing interest in the systemic diseases that may be caused by or associated with periodontal pathogens and inflammation. This topic was even the focus of the 9th European Workshop in Periodontology, which took place in 2012 (Linden et al., 2013). The proposed hypothesis for periodontal pathogens causing inflammation is two‐folds. First, bacteria and their products may enter the circulation through oral wounds caused by brushing, eating, or even dental treatment. They travel to other areas in the body and cause metastatic infection and inflammation. Second, the inflammatory mediators such as TNF‐α and IL‐1β produced locally in periodontal tissue may also enter the circulation and cause systemic or distant site inflammation (Van Dyke & van Winkelhoff, 2013).

A recent systematic review has reported consistent detection of periodontal bacteria DNA in coronary atherosclerotic plaque, with *P*. *gingivalis* and *A*. *actinomycetemcomitans* being most frequently detected (Joshi et al., 2019). These bacteria and their virulence factors can induce cardiovascular endothelial dysfunction through invasion and inflammation, which further favors atheroma formation (Sanz et al., 2020). Experiments on apolipoprotein E knockout mice have shown that *P*. *gingivalis* LPS‐induced inflammation is highly associated with atherosclerotic lesion progression. The bacterial heat shock protein GroEL of *P*. *gingivalis* also contributes to atherosclerosis through the up‐regulation of TLR4 (Huang et al., 2016). Furthermore, immune cross‐reaction to GroEL with the human heat shock protein hHSP60 expressed by arterial endothelial cells could also result in endothelial dysfunction. Periodontal pathogens and their virulence factors have also been found in the brain tissue of patients with Alzheimer's disease. Indeed, a connection between chronic periodontitis and cognitive impairment and Alzheimer's disease has been drawn after studies show a continuous pattern (Holmer et al., 2018). Inflammation induced by periodontal pathogens potentially alters the blood–brain barrier transport and accumulation of amyloid‐ß protein, a characteristic of Alzheimer's disease. Both LPS and gingipain of *P*. *gingivalis* induced inflammation in brain cells through upregulation of cytokines such as TNF‐α, IL‐1β, and IL‐6 and resulted in neurodegeneration or learning disability (Ilievski et al., 2018). *A*. *actinomycetemcomitans* cells also upregulate pro‐inflammatory cytokines and induce accumulation of amyloid‐ß peptides in brain cells through its LPS. Furthermore, they induce apoptosis and establish a pro‐inflammatory milieu in blood–brain barrier endothelial cells through leukotoxin (Díaz‐Zúñiga et al., 2019).

Another disease that has been linked with periodontitis is type 2 diabetes mellitus, in which the bi‐directional relationship has been well documented. *P*. *gingivalis* LPS has been shown to induce secretion of pro‐inflammatory adipokines leptin and resistin, IL‐6, and monocyte chemoattractant protein 1 (MCP‐1). It also promotes oxidative stress, which together may participate in obesity and insulin resistance (Le Sage et al., 2017). *P*. *gingivalis* inoculation led to more pro‐inflammatory cytokine production in diabetic mice compared to normal mice (Nishihara et al., 2009).

The role that periodontal bacteria may play in Rheumatoid Arthritis has also been investigated. *P*. *gingivalis* can citrullinate proteins through its virulence factors gingipain and PADs. The antibodies that target these citrullinated proteins may cross‐react with physiological citrullinated proteins, an autoimmune characterization of rheumatoid arthritis (Gómez‐Bañuelos, 2019).

Periodontal pathogens also play a role in adverse pregnancy outcomes, as *P*. *gingivalis* invasion of the amniotic cavity was found in women with threatened premature labor (León et al., 2007). *F*. *nucleatum* has been detected in chorion tissue from high‐risk pregnant women. Furthermore, LPS from *F*. *nucleatum* induces IL‐6 and corticotrophin‐releasing hormones in chorion‐derived cells, indicating that the pro‐inflammatory environment and dysregulated parturition condition induced by periodontal pathogens may lead to pregnancy complications (Tateishi et al., 2012).

Some of the other diseases that have been linked to periodontal disease but its relationship less studied include chronic obstructive pulmonary disease (COPD), pneumonia, and cancer. COPD is mainly caused by smoking but may be exacerbated by infection and inflammation of the lungs. A recent study demonstrated that periodontal pathogens, especially *F*. *nucleatum*, induced IL‐6 and IL‐8 in bronchial epithelial cells and IL‐8 in alveolar epithelial cells (Hayata et al., 2019). Another lung‐related disease, Pneumonia, is caused by infection of the lungs by a variety of microorganisms, including bacteria. A mice study showed that intratracheal inoculation of *P*. *gingivalis* led to lung damage accompanied by elevated levels of TNF‐α, IL‐6, IL‐17, and C‐reactive protein (CRP). These effects were dependent on the virulence factor gingipain, as inoculation with gingipain‐null *P*. *gingivalis* did not cause increased levels of IL‐6 and TNF‐α (Benedyk et al., 2016). Lastly, periodontitis has been associated with multiple types of cancer. Positive correlation between cyclooxygenase‐2 (COX‐2) products, which can be activated by periodontal bacteria‐induced cytokines, and the development of tumor and metastatic sites have been reported (Malinowski, 2019). Furthermore, other mechanisms linking the two diseases have been suggested. A study demonstrated that *P*. *gingivalis* GroEL enhances tumor growth by promoting the neovasculogenesis of tumor cells (Lin et al., 2015). Another study showed that *P*. *gingivalis* and *F*. *nucleatum* coinfection stimulated oral squamous cell carcinoma tumorigenesis via direct interaction with oral epithelial cells through TLRs (Gallimidi et al., 2015). It has also been suggested that tumor suppressor gene *p53* mutations, commonly detected in patients with pancreatic cancer, may be caused by the enzymes of periodontal bacteria (Öğrendik, 2016).

To make it easier to understand the mechanism of pathogen‐induced inflammation/diseases, we have organized the following into Table 1.

**Table 1:** Major oral bacteria‐induced inflammation/disease via activation of factors

Function	Secretion	Receptor	Activation/induction			
Pathway			Kinase	Factor	Cytokines and chemokine	Inflammation/disease
Oral bacterial						
P.g.	LPS	TLR2/4	ERK1/2 IRAK JNK PI3K P38 MAPK	MyD8u8 TRIF NF‐kB LITAF AP‐1	TNF‐α and IL‐1β IL‐2~6, IL‐8, IL‐10, CCL2,3, and ICAM‐1 VCAM‐1	Inflammation, neuro‐degenerative diseases, chronic diseases, atherosclerosis, Alzheimer's disease, Parkinson's disease, and cardiomyopathy
	Gingipain	TLR2/4	p38 EPK1/2	NFkB LITAF	TNF‐α and IL‐1β	Inflammation cancer
				MyD88	TNF‐α, IL‐6, IL‐10, IL‐23, and NO	Inflammation,
				p53, Bid		Apoptosis
	GroEL	TLR4 CXCR4 CCR5		PTX3 HSF13 LITAF	TNF‐α and IL‐β	Parkinson's disease and Alzheimer's disease
	PGN	TLR2		MyD88	TNF‐α and IL‐23	Chronic disease
T.f.	LPS and BspA	TLR2/4 and NLRP10	PI3K and ERK1/2	NF‐kB	TNF‐α, IL‐1β, IL‐3, ‐5, ‐6, ‐8, ‐9, ‐12, IL‐17, MCP‐1, and RANTES	Atherosclerosis, esophageal cancer, rheumatoid arthritis
F.n.	LPS	TLR2/4	MAPK, ERK1/2, and JNK	MyD88 NF‐kB	TNF‐α, IL‐1β, IL‐6, ‐8, and CRH	Adverse pregnancy outcomes
	FadA	E‐Cadherin		LEF/TCF NF‐kB	IL‐6, ‐8, ‐10, ‐18, and IFNy	Cancer
T.d.	MSP	TLR2	ERK1/2 P38MAPK	MyD88 NF‐kB	TNF‐α	Atherosclerosis rheumatoid arthritis
	LOS	TLR4	MKK1‐3, MKK6	MyD88 NF‐kB	TNF‐α, NO, IL‐6, ‐8, MCP1 MMP‐3, and PGE2	
	PGN		ERK1/2 GRK2, Lyn		TNF‐α, IL‐1β, IL‐6, ‐8, MMP‐9 RANTES, and PGE2	
	Periplasmic Flagella	TLR2		NF‐kB	TNF‐α, IL‐1β, and IL‐6, ‐10, ‐12	
A.a.	LPS	TLR4	MK2, JNK P38MAPK	MyD88 NF‐kB,AP‐1	TNF‐α, IL‐6, MMP‐2/3, and TIMP‐1	Atherosclerosis
	LtxA	LFA‐1 and CD18		Caspase 1	IL‐1β, IL‐18, MMP‐8, ICAM‐1 and VCAM‐1	Rheumatoid arthritis
	CDT	NLRP3		Caspase 1	TNF‐α, IL‐1β, and IL‐6	Alzheimer's disease, nonalcohoklic fatty liver disease

## 5 INHIBITORS OF MAJOR PERIODONTAL BACTERIA INDUCED INFLAMMATION

As mentioned above, the prolonged inflammation caused by the host response to bacteria and their virulence factors lead to periodontal disease and is associated with other systemic diseases. However, down‐regulation of the inflammatory mediators induced by periodontal bacteria may result in inhibition or resolution of inflammation and subsequent disease. This can also be achieved through inhibitors. Many types of inhibitors have been proposed to be used in adjunct to periodontal treatment, but the only agent that has been approved by the United States Food and Drug Administration (US FDA) and used clinically so far is a submicrobial‐dose doxycycline, which functions by downregulating matrix metalloproteinases (MMPs) and mediators such as IL‐6, TNF‐α, and prostaglandin E2 (PGE2). This approach, termed as “host modulation therapy”, was initially discovered by Golub and colleagues in the 80s, when experiments showed that orally administered minocycline in diabetic rats resulted in inhibition of pathologically excessive collagenase activity in their gingival tissues (Golub et al., 1985). Nevertheless, many other anti‐inflammatory agents have and are still being examined for their effects against periodontal inflammation. Nonsteroidal anti‐inflammatory drugs (NSAIDs) initially showed promise in periodontal treatment (Salvi & Lang, 2005).

They downregulate inflammation by blocking the cyclooxygenase (COX enzymes), therefore regulating arachidonic acid metabolite prostaglandins. Their effects on the treatment of periodontal disease were widely researched starting in the early 80s (Das, 2005). Although non‐selective Nonsteroidal anti‐inflammatory drugs (NSAIDs) such as aspirin, flurbiprofen, ibuprofen, and COX‐2 selective NSAIDs such as celecoxib showed promise in both animal and human studies, as they helped stabilize periodontal conditions by reducing the rate of alveolar bone resorption (Salvi & Lang, 2005), they can also lead to gastrointestinal problems, decreased platelet aggregation, renal and hepatic impairment, and cardiovascular issues. Furthermore, it was later discovered that bone loss accelerates when NSAIDs are stopped abruptly due to the rebound effect. Therefore, the routine adjunct use of NSAIDs has been precluded from periodontal treatment. However, alternative methods of NSAID use in periodontal treatment are still being examined up until this day, as a recent study demonstrated that the cyclic regimen of diclofenac potassium (50 mg BID, use for 2 months, stop for 2 months, then use again for 2 months) after initial non‐surgical periodontal treatment suppressed the increase of gingival crevicular fluid (GCF) IL‐1β levels at 6 months compared to the control group that only took placebos. Significantly lower levels of Prostaglandin E2 (PGE2) were also found compared to the control group at 6 months. No adverse effects were detected during this time. However, some rebound effects of PGE2 levels were noticed during the 2 month cessation period (Oduncuoglu et al., 2018). Another strategy utilizing NSAID derivatives claims to achieve greater results with reduced side effects. This rat study demonstrated that a hydrogen sulfide‐releasing derivative of the NSAID drug ketoprofen reduced expressions of LPS‐induced IL‐1β, TNF‐α, COX‐2, and inducible nitric oxide synthase (iNOS) and also showed less gastric damage compared to ketoprofen. However, the rebound effect was not addressed (Gugliandolo et al., 2018). Further studies will be needed to clarify the practicality of these new methods.

Another type of promising anti‐inflammatory therapy that has yet to find its way into routine periodontal treatment is anti‐cytokine agents, despite widespread use in treating cancer and various autoimmune diseases such as rheumatoid arthritis and psoriasis. Cytokines are cell‐signaling molecules that can either be pro‐inflammatory or anti‐inflammatory. Agents that aim to down‐regulate pro‐inflammatory cytokines or up‐regulate anti‐inflammatory cytokines have the potential to significantly reduce inflammation. They achieve down‐regulation of pro‐inflammatory cytokines through utilizing antibodies against specific cytokines, blocking cytokine receptors with cytokine antagonists, using soluble cytokine receptors to block its action, or disrupting the upstream signaling pathways of the cytokines (Lai & Dong, 2016). The drug infliximab, a neutralizing antibody to TNF‐α used in the treatment of rheumatoid arthritis among other autoimmune diseases, has been evaluated for its effect against periodontal disease in both animals and humans. Infliximab showed significant anti‐inflammatory and bone‐protective effects in a Wistar rat model with experimentally induced periodontitis, as it reduced gingival IL‐1β, TNF‐α, and myeloperoxidase levels, and also diminished MMP‐1/‐8, receptor activator of nuclear factor κB (RANK), and RANK‐Ligand (RANKL) bone immunolabeling compared with the control group (Gonçalves et al., 2014). Studies also evaluated the effect of Infliximab on rheumatoid arthritis patients with co‐existing periodontitis and noticed that they exhibited decreased periodontal attachment loss, and had lower periodontal indices and TNF‐α gingival crevicular fluid levels (Mayer et al., 2009). Soluble cytokine receptors have also been studied for its effect against periodontal disease. Etanercept, a recombinant soluble receptor to TNF‐α that is used for treating autoimmune diseases, led to significantly reduced periodontal inflammation, infiltration of neutrophils, and iNOS levels in rats with experimentally induced periodontitis (Di Paola et al., 2007). Other soluble receptors or antagonists to IL‐1 or TNF‐α were used in monkey models with experimentally induced periodontitis and either showed a significant reduction of inflammatory cell infiltrates, reduction of osteoclast numbers, or reduced attachment and bone loss (Graves et al., 1998). Manipulation of the signaling pathways of LPS‐induced cytokines has been explored, as inhibition of p38‐α mitogen‐activated protein kinase (p38 MAPK) resulted in a significant reduction of TNF‐α, IL‐1, and IL‐6 (Kirkwood & Rossa, Jr., 2009). The main concern of anti‐cytokine therapy is that the down‐regulation of the immune system increases the risk of infection. For instance, an opportunistic infection has been reported when TNF‐α was neutralized for rheumatoid arthritis therapy (Keane et al., 2001). Therefore, these safety issues need to be resolved before anti‐cytokine therapy can be applied in the field of periodontal treatment.

In addition to anti‐cytokine therapy, which either blocks the cytokines from functioning or inhibits the signaling pathways of cytokines, cutting‐edge technology known as RNA interference (RNAi) can inhibit gene expression or translation. Initially observed in the early 1990s, Fire and Mello further discovered that RNAi functioned through double‐stranded RNA, which eventually won them a nobel prize in physiology or medicine in 2006. This technology utilizes small RNAs, which in its mature form can bind to messenger RNA (mRNA) and cleave them through the RNA‐induced silencing complex (RISC), therefore knocking down the expression of target proteins. RNAi can be further subdivided into small interfering RNA (siRNA), short hairpin RNA (shRNA), and microRNA (miRNA). Various methods to deliver RNAi have been examined, including viral and bacterial vectors, transfection, and carriers such as chitosan. The first RNAi‐based therapy was approved by the US FDA in August 2018 to treat the genetic disease hereditary transthyretin‐mediated amyloidosis (Kuehn, 2018). Additionally, many other therapeutic applications are being examined, including its use in periodontal disease. A study demonstrated that knockdown of RANK in RAW 264.7 cells and primary bone marrow cells by siRNA resulted in short term repression of Receptor activator of nuclear factor κB (RANK) expression without off‐targeting effects, and significantly inhibited both osteoclast formation and bone resorption (Wang & Grainger, 2010). In terms of inhibiting oral bacteria‐induced inflammation, another study demonstrated that utilizing siRNAs against MAP kinase‐activated protein kinase 2 (MK2) in rat bone marrow stromal cells resulted in inhibition of *A*. *actinomycetemcomitans* LPS ‐ induced IL‐6 and TNF‐α production and COX‐2, IL‐1β, and chemokine (C‐X‐C motif) ligand 1 (CXCL1) mRNA levels. Furthermore, it arrested *A.actinomycetemcomitans* LPS‐induced inflammatory bone loss and decreased inflammatory infiltrate and osteoclastogenesis in a rat model (Li et al., 2011). Other reports have utilized RNAi knockdown against gene expression of Cathepsin K, TNF‐α, and heme oxygenase‐1. Collectively, these small RNAs can inhibit inflammatory mediators and reduce alveolar bone and periodontal tissue loss (Tenkumo et al., 2020).

An exciting discovery of “specialized pro‐resolution mediators” has brought a new concept into the battle against periodontal inflammation. In the past, resolution of inflammation was thought to be a passive process, where pro‐inflammatory mediators dissipate after serving their purpose. However, Charles Serhan's group discovered that neutrophils can metabolize arachidonic acid and other polyunsaturated fatty acids (PUFAs) into pro‐resolution lipid mediators of inflammation. These mediators, which function to actively resolve inflammation through counteracting the actions of pro‐inflammatory signals such as prostaglandins and leukotrienes, were later classified into lipoids, resolvins, maresins, and protectins among others. These resolution mediators were brought into the field of research in periodontology in the early 2000s and has steadily gained popularity as they showed great promise in animal experiments (Serhan et al., 2003). A recent systematic review of specialized pro‐resolving lipid mediators in experimental periodontitis suggested that they can prevent alveolar bone loss, regenerate bone, and induce a positive shift in microbial composition and inflammatory status when applied topically (Osorio Parra et al., 2019).

Another trending anti‐inflammatory agent is natural compounds, which are considered relatively safe and inexpensive. They have long been an essential part of traditional medicine in many countries including China, Korea, and Japan. Most of them were developed through trial and error, although many of them are now being tested through evidence‐based methods. For example, kavain is a lactone found in kava plants. Along with its analogs Kav001, it has shown anti‐inflammatory and anticonvulsive properties. Several studies demonstrated that they reduced LPS‐induced pro‐inflammatory cytokines in‐vitro and also exhibited significant anti‐inflammatory effects in animals (Tang et al., 2018). Another example of a natural compound is curcumin, a biologically active polyphenolic compound found in turmeric, an Indian spice derived from the plant *Curcuma longa* Linn. It exhibits anti‐inflammatory, anti‐oxidant, and anti‐bacterial effects among other properties. Studies have shown that they inhibit pro‐inflammatory cytokines including IL‐1β, TNF‐α, and IL‐6 through blocking signaling pathways of NF‐κB and p38 MAPK (Zambrano et al., 2018). Furthermore, animal experiments have demonstrated that curcumin inhibits inflammatory bone resorption, decreases osteoclast counts, and inflammatory infiltrates, and reduces MMPs production (Guimarães et al., 2011). Human studies have shown that the adjunctive use of curcumin with scaling and root planing (SRP) resulted in significant improvement of periodontal parameters compared to SRP alone (Raghava et al., 2019). Furthermore, in order to increase its MMP inhibiting potency, a chemically modified curcumin, CMC2.24 has been shown to be effective in reducing periodontal inflammation and MMP production through inhibition of signaling molecules TLR‐2 and p38 MAPK (Deng et al., 2020).

Studies have also examined methods to suppress TLR signaling, including inhibiting TIRAP, inhibiting MyD88, blocking NFkB, or competing with CD14 and LBP (LPS binding protein). Chloroquine, an antimalarial agent that blocks TLR‐9 signaling, has been shown to significantly decrease inflammatory cytokines induced by periodontal pathogens (Sahingur et al., 2012). Female sex hormones β‐estradiol and progesterone both significantly reduced *P*. *gingivalis* LPS‐induced TLR2 expression (Jitprasertwong et al., 2016). Lastly, many attempts have been made to inhibit specific periodontal pathogens and their virulence factors. Chlorhexidine, polyphenols, and some protein‐derived peptides are among agents that inhibit gingipain (Olsen & Potempa, 2014). The antimicrobial peptide LL‐37 is effective against *T*. *denticola* and human saliva inhibits dentisilin (Rosen et al., 2012). Some bacteria from subgingival flora can inhibit *A.actinomycetemcomitans* by degrading its leukotoxin (Johansson et al., 2000).

To make it easier to search and analyze the information about the inhibitors treated for the pathogen‐induced diseases, we have organized the following into Tables 2.

**Table 2:** Major inhibitors in response to oral bacteria treatment

Function	Reduction/inhibition			
Object	Kinase/factors	Cytokine/chemokine	In vitro treatment	In vivo treatment
Name of inhibitor				
MMG11, Pam3CSK4, O‐Vanillin	TLR2		Interaction with receptor	
TAK‐242, E6446, EM‐163, Melatonin	TLR4		Interaction with receptor	
Angiotensin II receptor blocker	TNF‐α and IL‐1β			
anti‐HMGB1 Ab	IL‐1β and CXCL1			Neutrophil recruitment
Antimicrobial peptide	IL‐1β and TNF‐α		Alveolar bone loss	
Azithromycin	Rac1 and NF‐κB	IL‐6, ‐8, MCP‐1, and GRO	Probing depth and increased periodontal attachment	
18beta‐GA	NF‐κB	IL‐1β, and IL‐12p70	Alveolar boneloss	
Baricitinub inh	JAK	Cytokines		
Bisphosphonate		Cytokine production	Osteoclast activity	
Cyclosporine‐A	MMP			
Cystatins	Cysteine peptidases	Cathepsin B and K, IL‐1β, and TNF‐α		
Dimetyloxallyl glycine		IL‐6, ‐8, TNF‐α, and IL‐1β		
DX‐9065a	Coagulation factor X.NF‐κB	IL‐6		
Electro acupuncture		IL‐1β and MMP‐8	Alveolar boneloss	
Fluoxetine		IL‐1β, COX‐2, and MMP‐9		
EMD		TNF‐α	Inflammation	
Etanercept		TNF‐α		Periodontal inflammation
Glycoside		Myeloperoxidase, IL‐1β, and TNF‐α	Inflammation	Insulin
Grape seed proanthocyanidin extracts			LPS‐induced inflammation	
HDACs inh		IL1‐B, CCL2, CCL5, CXCL10, COX2, MMP3	Alveolar boneloss	
HO‐1 inh	Heme oxygenase‐1, nitric oxide, and PGE2			
4‐Hydroxycordoin	NF‐κB and AP‐1	IL‐1β, TNF‐α, IL‐6, ‐8, CCL5, and PGE2		
Infliximab		TNF‐α		Clinical attachment loss
LED irradiation	MAPK	IL‐6 and IL‐8		
Lindenenyl acetate	HO‐1, AMPK, JNK MAPK, and Nrf‐2	PGE2, TNF‐α, IL‐1β, IL‐6, and IL‐12		
5‐lipoxygenase inh	5‐lipoxygenase	TNF‐α and IL‐12	Bone resorption	
Luteolin	iNOS and RANKL		Alveolar boneloss	
Melatonin		TNF‐α, IL‐1β, and MCP‐1	Probing depth	Enhanced bone repair
Methyl gallate and gallic acid	*Fusobacterium nucleatum*	IL‐6 and IL‐8		
ML324		TNF‐α and IL‐6		
MSE		MMP‐1, MMP‐9, and TIMP‐1		
NOS inh	Nitric oxide synthase		Alveolar boneloss	
0.1% OLG		IL‐8, CCL20, and GRO‐α		
Polyphenols	TLR2/4, MyD88, NFκB, and PPARγ	MCP‐1, PAI‐1, and IL‐6		
Prenylated flavonoids				Growth of P.g
Quercetin	TLR4 and NF‐κB	IL‐1β, IL‐6, and IL‐8		
Resistin inh	COX‐2, iNOS, TNF‐α, IL‐1β, ‐6, ‐12, and MMPs	Resistin and adipocytokine	Insulin resistance	
Resveratrol	Cox2 and Nf‐κb			
SD‐282	p38 MAPK		Alveolar boneloss	
Sirt1	TLR4 and JNK/NF‐κB	IL‐1α, IL‐6, ‐8, and TNF‐α		
Statins	HMG‐CoA reductase	IL‐1β, MMP‐2, ‐9, RANK, RANKL, COX‐2, and TNF‐α	Alveolar boneloss	
1,25‐dihydroxy vitamin D3	AhR/NF‐κB	IL‐6		
Submicrobial‐dose doxycycline		Cytokines, MMPs	Pocket depth	
Theaflavins		IL‐1β, TNF‐α, IL‐6, CXCL8, and MMP‐3, ‐8, ‐9		
TNF and IL‐1 antagonist		TNF‐α and IL‐1	Alveolar boneloss	
Tormentic acid	TLR4, NF‐κB, IκBα, and ERK.JNK.P38	IL‐6 and IL‐8		
UDCA		IL‐1α, IL‐1β, and IL‐6		

## 6 CONCLUSION

Evidence supports a strong association between periodontal disease and various systemic diseases; this link may have direct and indirect effects of periodontal pathogens. Although we investigated how periodontal bacteria and their virulence factors can trigger host immune defense and induce many systemic diseases via inflammation and invasion, further studies will be needed to identify the causative mediators of these association. This review also discusses the latest research regarding inhibitors against inflammation, how the downregulation of the inflammatory mediators induced by periodontal bacteria may reduce the likelihood or result in a resolution of periodontitis and periodontal‐related systemic diseases, and the effectiveness of clinical therapy for many patients. Overall, this review will prove to be a helpful reference for researchers that summarizes the past, present, and future beliefs and treatments.

## CONFLICT OF INTEREST

Non‐conflict of interest in the manuscript.

## AUTHOR CONTRIBUTIONS

Xiaoren Tang and Serge Dibart designed and instructed this study; Xiaoren Tang, Thuraya Elgreu, Sean Lee, and Sabrina Wen drafted the manuscript; Xiaoren Tang, Thuraya Elgreu, Sean Lee, Radwa Elghadafi, Thanarut Tangkham, Yun Ma, and Bing Liu analyzed the data of the manuscript; All authors revised the manuscript and gave final approval and agree to be accountable for all aspects of the work.

## Data Availability

Data sharing not applicable ‐ no new data generated, or the article describes entirely theoretical research. Data sharing not applicable to this article as no datasets were generated or analysed during the current study.
